# Effect of an In-Office Bleaching Agent with Surface Pre-Reacted Glass-Ionomer Filler on the Enamel Surface: A In-Vitro Study

**DOI:** 10.3390/jfb14070386

**Published:** 2023-07-21

**Authors:** Mika Shimojima, Noriko Hiraishi, Kodai Akabane, Mohannad Nassar, Masayuki Otsuki, Yasushi Shimada

**Affiliations:** 1 Department of Cariology and Operative Dentistry, Tokyo Medical and Dental University, Tokyo 113-8549, Japan; 88pancho64@gmail.com (M.S.); akabane0106@gmail.com (K.A.); otsuki.ope@tmd.ac.jp (M.O.); simaodnt@tmd.ac.jp (Y.S.); 2 Department of Preventive and Restorative Dentistry, College of Dental Medicine, University of Sharjah, Sharjah P.O. Box 27272, United Arab Emirates; nassarmhnd@gmail.com

**Keywords:** surface pre-reacted glass-ionomer (S-PRG), H_2_O_2_-based bleaching agents, enamel erosion, surface roughness, microhardness, scanning electron microscope (SEM), energy dispersive X-ray (EDX)

## Abstract

In-office bleaching with high concentrations of hydrogen peroxide (H_2_O_2_) agents causes undesirable alterations in the enamel. Surface pre-reacted glass-ionomer (S-PRG) filler is a functional material known for its acid-neutralizing and demineralization-inhibition properties. This study evaluates the effect of S-PRG filler incorporation in H_2_O_2_-based bleaching on the enamel surface. Bovine enamel surfaces were bleached using a bleaching paste formulated with a liquid (35% H_2_O_2_) and a powder containing 5% or 10% S-PRG filler. The surface roughness and the Vickers microhardness of the treated enamel surfaces were evaluated. The enamel surfaces were observed under a scanning electron microscope (SEM) and analyzed using energy dispersive X-ray (EDX) technology. The surfaces were challenged by citric acid and observed by SEM. The specimens bleached with the paste containing the S-PRG filler showed lower enamel surface roughness and higher microhardness values than did those bleached with the plain paste (0% S-PRG filler); meanwhile, there were no significant differences between the 5% or 10% S-PRG filler groups. The S-PRG filler groups showed enamel surface morphologies similar to those of the non-bleached enamel, according to SEM observation, and EDX analysis detected the presence of fluoride and strontium ions. The S-PRG filler groups showed a higher resistance to erosion. The S-PRG filler mitigated the detrimental effects of bleaching agents on the enamel surface and provided resistance to erosion.

## 1. Introduction

Teeth discoloration is a prevalent problem among patients of different ages. Alteration in the color of teeth can be intrinsic or extrinsic in nature. Intrinsic discoloration occurs within the structure of the tooth, and its etiology is multifactorial, including metabolic diseases, infections, and medications, or it can be associated with aging, while extrinsic stains, which usually appear on the outer surface of the tooth, are mainly due to local factors such as smoking, dyes in food and beverages, and oral chromogenic bacteria accompanied by poor oral hygiene. However, certain chronic extrinsic stains that remain on the tooth surface for a long period of time develop into intrinsic stains [1,2].

Tooth bleaching is a common procedure performed in dental offices to improve aesthetics, and it is highly requested by patients who seek treatment to have discolorations removed. Hydrogen peroxide (H_2_O_2_) is the most-used agent for this purpose, which is manufactured in low concentrations for home use and high concentrations for in-office bleaching [3]. H_2_O_2_ is an unstable oxidizing agent affected by incident light, pH, temperature, and interactions with transition metals, resulting in a series of reactions, followed by its decomposition [4,5]. The bleaching efficacy of H_2_O_2_ relies on the generation of free radicals, which are highly reactive oxidants with unpaired electrons in their structure. These are capable of oxidizing organic pigments or chromogens and breaking them into small compounds with a diminished ability to absorb light and which can diffuse out of the tooth, thus creating the whitening effect [6,7,8].

The formation of different radical species from H_2_O_2_ depends on the pH and activation method [9]. The direct influence of the pH on the bleaching effect is well documented in the industrial field, in which H_2_O_2_ with higher pH values is used to enhance the bleaching effect on cotton and wood pulp. In contrast, most dental bleaching products are supplied as acidic gels to extend the shelf-life because H_2_O_2_ is more stable in acidic environments. The average pH of commercially available in-office bleaching gels is reported to be about 5.5, while there are many bleaching gels available with pH values as low as 3.6 to 6.5 [10]. However, the low pH can potentially cause unwanted consequences to the tooth structure, such as enamel demineralization and changes in its properties, composition, and surface morphology [11,12,13,14]. To overcome these problems, recent studies have evaluated the properties of dental bleaching agents at a neutrally adjusted pH. The findings regarding their enhanced bleaching efficacy and simultaneous mitigation of the adverse effects on enamel characteristics were promising [15,16]. Therefore, adjusting the pH to near neutral condition is crucial in developing new formulations of bleaching products. Wijetunga et al. used Na_2_CO_3_ to adjust the pH of bleaching agents [14], and Xu et al. adjusted the pH from 3 to 8 by adding 1 M HCl or 1 M NaOH solution to the H_2_O_2_ solution [16]. The whitening effect was enhanced by increasing the pH value, and no obvious morphological or chemical compositional changes in the enamel surface were detected in the bleaching solution in the neutral range [16].

In order to inhibit the demineralization and remineralization of the bleached tooth structure, fluoride was added to the bleaching agents [17,18]. However, these studies reported that the addition of fluoride failed to strengthen bleached teeth. Another approach included the incorporation of 45S5 bioactive glass into the H_2_O_2_ solution [19,20]. The H_2_O_2_ solution containing bioactive glass was effective for teeth whitening, increased the pH, inhibited enamel surface demineralization, and maintained the surface morphology. The functional materials, such as bioactive glass, used to adjust the pH value of H_2_O_2_ solutions are promising candidates for minimizing H_2_O_2_-induced enamel surface damage.

Surface pre-reacted glass-ionomer (S-PRG) filler represents a bio-functional material that releases various ions, namely fluoride, strontium, borate, aluminum, silicate, and sodium, that possess desirable acid buffering properties and contribute to the protection of the tooth structure through inhibiting demineralization and plaque formation and inducing remineralization [21,22]. S-PRG filler has a three-layered structure based on a fluoroboro-aluminosilicate glass core. It consists of a silicon dioxide (SiO_2_) coating on the outer layer, a pre-reacted glass ionomer phase underneath, and a glass core within. The glass ionomer phase is formed by spraying polyacrylic acid, penetrating the SiO_2_ coating layer, and reacting with the fluoroboro-aluminosilicate glass core in an acid-base reaction [23].

Fluoride and strontium ions can form an acid-resistant layer and reinforce tooth structure by acting on the hydroxyapatite to convert it to fluorapatite and strontium-apatite, respectively, and strontium, sodium, and boron can modulate the pH of the surrounding environment to a neutral or alkaline condition [24,25,26]. Thus, the S-PRG filler’s unique properties make the material well suited to several applications in dentistry, and the filler is now incorporated into resin-based restorative materials, dental adhesives, and sealants [21,22]. In addition, S-PRG filler has been experimentally incorporated into inorganic cements, root canal sealers, denture bases, tissue conditioners, and toothpastes [23]. However, as yet, there are still no reports evaluating the effect of S-PRG filler on enamel when incorporated into H_2_O_2_--based office bleaching materials.

Considering the growing attention that S-PRG filler has been generating and the acid buffering ability and protective properties of the released ions, it seems reasonably plausible to examine the effects on tooth enamel characteristics of the addition of S-PRG filler to bleaching products. We have previously evaluated the whitening efficacy of a bleaching agent containing S-PRG filler and reported that S-PRG filler improved the bleaching effect, increased the reaction rate, and elevated the pH value close to neutral [27].

In this study, S-PRG filler was blended with a professionally applied bleaching material (Shofu Hi–Lite, Shofu Inc., Kyoto, Japan), and changes in enamel roughness, hardness, morphological characteristics, and resistance to erosion were examined. The null hypothesis tested was that bleaching agents containing S-PRG filler would show no difference in their effects on enamel surface properties compared to the effects of those without the filler.

## 2. Materials and Methods

### 2.1. Specimen Preparation and Bleaching Procedure

**Figure 1:**
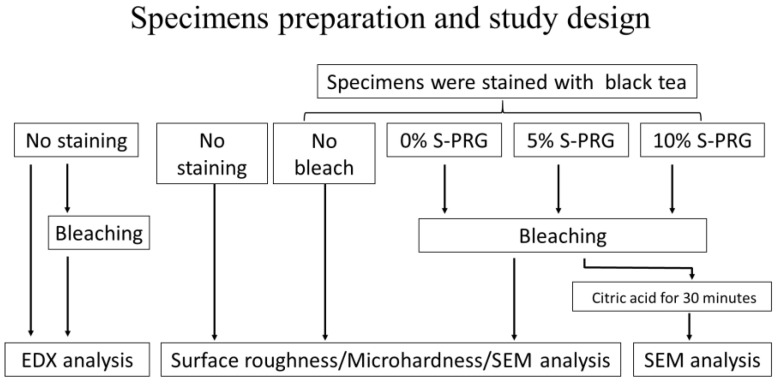
Specimen preparation and study design.

Figure 1 illustrates the specimen preparation. Bovine incisors were obtained as discarded specimens in an authorized procedure approved by the Food Safety Commission of Japan, Ministry of Health, Labor, and Welfare. The minimum number of samples for each experiment was determined by a pilot study and based on a power calculation of 0.8, with a 95% confidence level. The roots were removed, and soft tissue remnants attached to the teeth were cleaned gently with a scalpel. The labial enamel surface was ground with 600-grit silicon carbide paper (Sankyo Rikagaku Co., Ltd., Saitama, Japan) under running water until reaching approximately 1 mm thickness of the remaining enamel, which was measured by a digital caliper (Mitutoyo Corporation, Kanagawa, Japan). Two 5 × 5 mm specimens were obtained from the flattened labial surface of each tooth using an Isomet saw (ISOMET Buehler, Lake Bluff, IL, USA). The dentin surface of each specimen was covered with double-sided tape (Nice Tack, Nichiban, Tokyo, Japan), and each specimen was embedded in a cylindrical acrylic tube (10 mm in height and 10 mm in internal diameter) using dental self-curing acrylic resin (Unifast III, GC Corporation, Tokyo, Japan). Upon the setting of the resin, both sides of the specimen (enamel and pulpal surface of the dentin) were re-exposed by trimming away the excess resin. The tape was removed, and the dentin surface was irrigated with 5% sodium hypochlorite solution (Wako Pure Chemical Industries, Osaka, Japan) for 1 min to remove organic tissue remnants, followed by etching with 40% phosphoric acid (K-etchant gel, Kuraray Noritake Dental, Tokyo, Japan) for 10 s to open the dentinal tubules to enhance stain uptake into the specimen structure. The enamel surface of each specimen was polished with 1000- to 2000-grit silicon carbide paper to obtain smooth and flat enamel surfaces. Finally, the specimens were cleaned with an ultrasonic cleaner (US-2KS, SND, Nagano, Japan) for 5 min.

A staining process was performed to selected enamel specimens. Artificial staining was performed using black tea extract. The tea solution was prepared by immersing two tea bags (Lipton yellow label tea bags, Unilever Japan, Tokyo, Japan) in 50 mL of fresh boiling water for 5 min. The specimens were immersed in the tea extract and stored in an incubator for 7 days at 37 °C. The solution was stirred once a day to avoid precipitation in the solution and changed on the fourth day of the experiment. After removal from the solution, the specimens were rinsed under tap water and air-dried. The color of each enamel surface was measured with a colorimeter (NR-11A, Nippon Denshoku, Tokyo, Japan). The International Commission on Illumination (CIE) L* a* b* values were recorded. Specimen selection was based on the L* value, and only those with an L* value between 40–50 were selected in order to minimize variations among the specimens [27].

Table 1 lists the materials used in this study. The in-office tooth bleaching product (Shofu Hi–Lite, Shofu Inc, Kyoto, Japan) used is an H_2_O_2_-based agent sold as separate powder and liquid parts, which are mixed before use. S-PRG filler (lot. BFFG-412) was provided by Shofu Inc. (Kyoto, Japan) and formulated at 5 wt% and 10 wt% into the bleaching powder of the experimental groups. The specimens were randomly assigned to five groups (*n* = 8) according to the type of surface treatment and bleaching protocol used:

- The no staining group— enamel that were not stained.
- The no bleaching group—enamel surfaces unbleached after staining.
- The control (0% S-PRG) group—stained enamel bleached with plain paste without S-PRG filler.
- The 5% S-PRG group—stained enamel bleached with a paste containing 5% S-PRG filler in the powder.
- The 10% S-PRG group—stained enamel bleached with a paste containing 10% S-PRG filler in the powder.

The bleaching procedure was performed following the manufacturer’s instructions. One scoop of the powder and three drops of the liquid were mixed for 30 s using a spatula until a uniform paste was formed. The resultant paste was applied at a prescribed thickness onto the enamel surface. The paste was left undisturbed on the surface for 5 min, and then the sample was irradiated for 3 min using an LED light unit for tooth bleaching (G-Light Prima Ⅱ Plus, GC Corporation, Tokyo, Japan) at a peak wavelength of around 400 nm. The paste was left on the surface for another 2 min and then removed with damp cotton. The specimens were washed thoroughly under tap water and dried gently. This bleaching process was repeated three times on each specimen.

### 2.2. Assessment of Surface Roughness 

Using a confocal laser microscope (CLSM; VK-X 150 series, Keyence, Osaka, Japan), the specimens were subjected to enamel surface roughness evaluation at 20× magnification. Sa (arithmetical mean height of area) was used as a roughness parameter. The Sa is the extension of Ra (arithmetical mean height of a line) to a surface. It expresses an absolute value of the difference in height of each point compared to the arithmetical mean of the surface. The Sa (μm) is defined as

$$\mathrm{Sa}=\frac{1}{A}\iint_{A}^{k}\left|Z\left(x,y\right)\right|dxdy$$

where *A* = the defined area, *Z* = the absolute value of the height of the points, and *x,y* = the measurement unit of the XY stage. The Sa measurement used in this study is the ISO 25178 surface texture parameter. For the Sa measurement, the S filter nesting index was set to none, and the L filter nesting index was set to 200 μm. The Sa was measured before the bleaching procedure, for baseline data, and again after completing the three bleaching cycles. Each specimen (5 mm square) was divided into four equal parts (2.5 mm square) on the image, and an area of 100 μm square was set at the center of each part, and its Sa was measured. Then, the average value of Sa measured four times per surface was defined as the surface roughness of the sample to achieve objective measurement.

### 2.3. Assessment of Microhardness

The enamel specimens used for surface roughness were employed for microhardness evaluation because the CLSM allows Sa values to be measured without contact with the surface, so the enamel surface remains intact. Vickers microhardness (VHN) measurements were performed, before and after bleaching, on each specimen using a microhardness tester (HM-102, Mitutoyo Corporation, Kanagawa, Japan). For each specimen (5 mm square), the surface was divided into four equal sections (2.5 mm square), and an indentation was created in the center of each divided section of the enamel surface. The measurement load was 100 g, and the dwell time was 15 s. The average of those four values was obtained in the same manner used for the surface roughness measurement.

### 2.4. Assessment of Morphological Changes

The same specimens used for the roughness and hardness tests were used for this part of the study. The surface morphology of the bleached enamel surfaces was evaluated and compared with the unbleached enamel surface. The enamel specimens were sputter-coated with gold and observed by scanning electron microscopy (SEM; JSM-IT 100, JEOL, Tokyo, Japan) at 2000× magnification under 15 kV operating conditions.

### 2.5. Energy Dispersive X-ray (EDX) Analysis

Specimens (5 mm squire) were prepared in the same manner described above; however, the staining process was not conducted, in order to avoid any influence of the tea components on the analysis. The 32 specimens were randomly divided into four groups (*n* = 8). The enamel surfaces were prepared by carbon coating. The center of the prepared specimen was observed by SEM at a magnification of 500 under 15 kV operating conditions and analyzed with an EDX to identify the elemental composition. Eight qualitative elements, B, F, Al, Si, P, Ca, Mn, and Sr, were selected, and the relative quantitative values (atom %) for each qualitative element were calculated. The elements of F and Sr were used to compare the effects of the experimental bleaching pastes on the enamel surfaces.

### 2.6. Assessment of Erosion by Citric Acid

Twenty-four enamel specimens were prepared in the same manner as that described above and were randomly divided into three groups: bleaching with plain paste (0% S-PRG filler) and bleaching with the paste containing 5% and 10% S-PRG filler in the powder portion. Each specimen was immersed in 10 mL of 1% citric acid (pH 2.12) for 30 min at 37 °C. After acid challenging, the enamel surfaces were sputter-coated with gold and observed by SEM at 2000× magnification under 15 kV operating conditions.

### 2.7. Statistical Analysis

One-way ANOVA was applied to the surface roughness, microhardness, and EDX data, followed by a Tukey’s HSD post hoc test, with a 5% significance level. All statistical analyses were performed using statistical software (SPSS ver. 27.0 for Windows, IBM, Chicago, IL, USA).

## 3. Results

### 3.1. Surface Roughness

**Figure 2:**
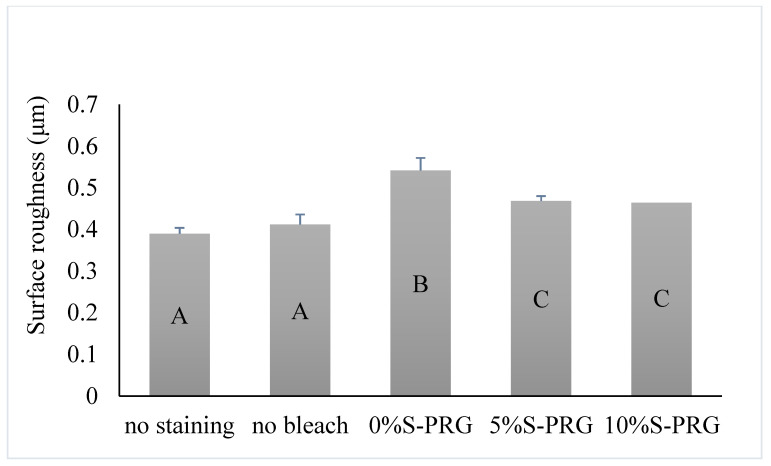
Means and standard deviations for the surface roughness (Sa) of bovine enamel after different treatment protocols. Identical letters indicate no significant difference (*p* > 0.05).

Figure 2 shows the mean and standard deviation (SD) of the Sa of the tested groups. One-way ANOVA revealed a significant difference in Sa between the groups (*p* < 0.001). The 0% S-PRG group had a significantly higher mean value of Sa than the other groups (*p* < 0.001), indicating a greater surface roughness. Specimens bleached with the paste containing S-PRG filler (5% and 10% S-PRG groups) had a higher mean value of Sa than did the enamel surfaces without staining, as well as the no bleach groups (*p* < 0.05). However, no significant differences existed between the 5% S-PRG and 10% S-PRG groups (*p* = 0.995).

### 3.2. Microhardness

**Figure 3:**
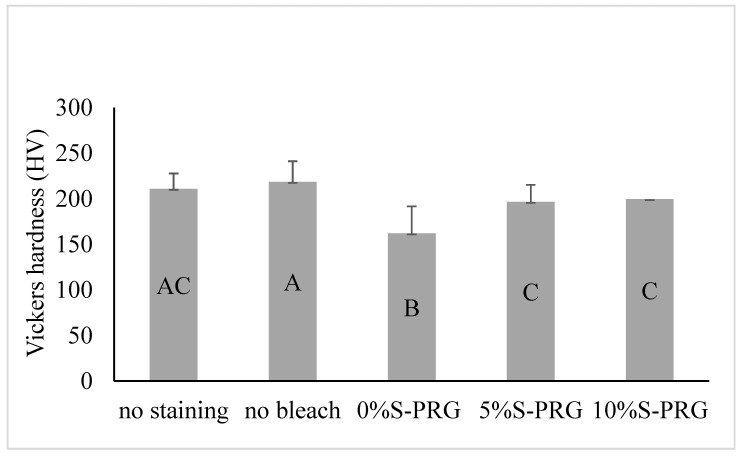
Means and standard deviations for microhardness of bovine enamel after different treatment protocols. Identical letters indicate no significant difference (*p* > 0.05).

Figure 3 illustrates the mean and SD of the VHN of the tested groups. One-way ANOVA revealed a significant difference in VHN values between the groups (*p* < 0.001). The 0% S-PRG group had a significantly lower mean value of VHN than the other groups (*p* < 0.001). Specimens bleached with the paste containing S-PRG filler (5% and 10% S-PRG groups) had a lower mean value of VHN than did the no bleach group (*p* < 0.05). However, no significant differences existed between the 5% S-PRG, 10% S-PRG, and enamel surfaces without staining groups (*p* = 0.192).

### 3.3. Surface Morphology

**Figure 4:**
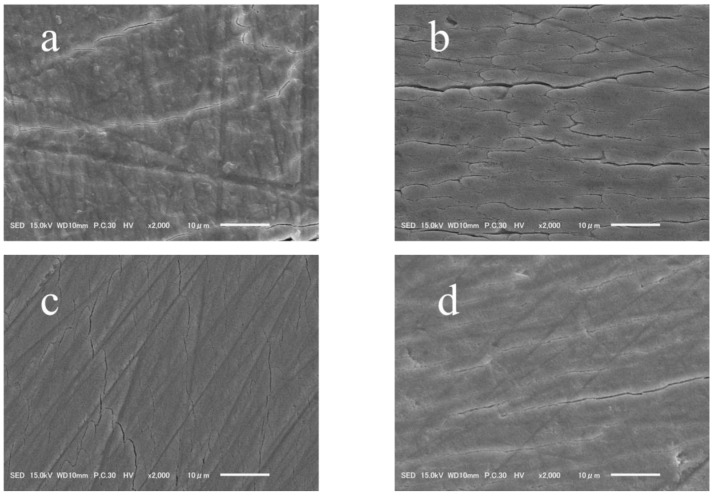
Representative scanning electron photomicrographs of enamel surfaces after the different treatment protocols. (**a**) No bleach: smear layer created by 600-grit silicon carbide paper covering the enamel surface. There are multi-directional scratches produced by polishing with silicon carbide paper. (**b**) Control group (bleached with 0% S-PRG filler): smear layer removal along with the disappearance of the scratches and exposure of the enamel rods beneath. These surface alterations are suggestive of changes in the mechanical properties of the substrate. (**c**,**d**) Experimental groups (bleaching paste with 5% or 10% S-PRG filler; respectively): smear layer created by 600-grit silicon carbide paper and multi-directional scratches. There are no exposed enamel rods.

Figure 4 presents the representative images of the morphological assessment under SEM. The unbleached enamel surfaces showed a smooth topography with uniform multidirectional scratches resulting from preparing the surface with 600-grit silicon carbide paper (Figure 4a). Meanwhile, the control group (0% S-PRG) specimens revealed the disappearance of most of the scratches, which reflects the removal of the smeared surface and the exposure of the enamel rods beneath (Figure 4b). However, specimens bleached with the paste containing S-PRG filler (5% and 10% S-PRG groups) showed surface morphologies with a smooth topography (Figure 4c,d).

### 3.4. EDX

**Figure 5:**
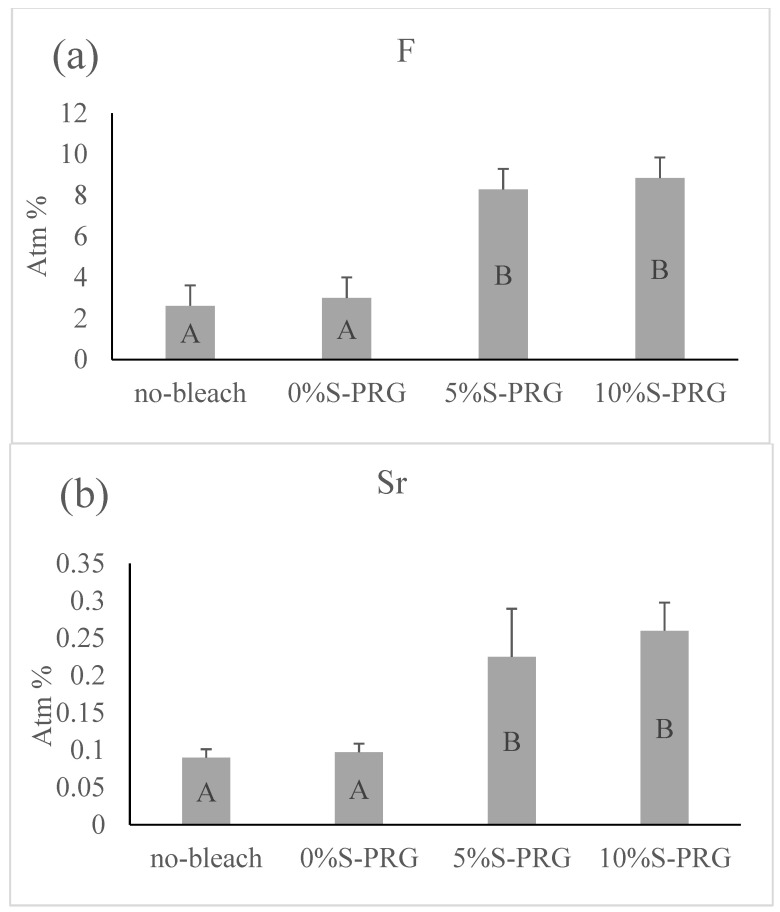
Means and standard deviations of (**a**) fluoride and (**b**) strontium ions detected by energy-dispersive X-ray spectroscopy of bovine enamel surfaces after the different treatment protocols. Identical letters indicate no significant difference (*p* > 0.05).

Figure 5 demonstrates the average amounts of fluoride and strontium ions detected on the surface of the specimens from each group. There was a statistically significant difference in the amount of fluoride and strontium between the control group and the S-PRG filler groups (*p* < 0.001). S-PRG filler groups showed higher amounts of these elements than did the other groups, but fluoride and strontium were *p* = 0.128 and *p* = 0.069, respectively, for the 5% S-PRG group, and there was no significant difference between the 5% S-PRG group and the 10% S-PRG group. For the non-bleached groups, the levels of fluoride and strontium were comparable to those of the control group (0% S-PRG), with *p* = 0.992 and *p* = 0.402, respectively.

### 3.5. Erosion by Citric Acid

**Figure 6:**
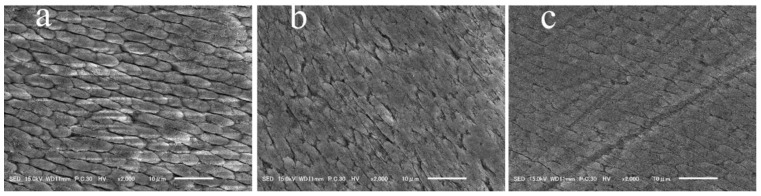
Representative scanning electron photomicrographs of enamel surfaces of bleached enamel specimens after a 30 min acidic challenge with citric acid: (**a**) 0% S-PRG in the bleaching paste and (**b**,**c**) 5% and 10% S-PRG in the bleaching paste, respectively.

Figure 6 includes representative images of the morphological evaluation under SEM. The control group’s specimens (0% S-PRG) exhibited the exposed patterns of the underlying enamel rods, indicating an irregular surface texture (Figure 6a). However, the enamel surfaces were smoother in the samples bleached with the paste containing S-PRG filler (Figure 6b,c) than in those of the control group (Figure 6a).

## 4. Discussion

The increased attention to aesthetic dentistry, including white teeth, has created a surge in the demand for professional bleaching products. These products are mostly acidic and contain high concentrations of H_2_O_2_ [10], which can have deleterious effects on enamel surfaces; thus, new formulations of bleaching products are constantly emerging in the market with the claim of having a milder effect on the tooth structure. In the current investigation, the effect of S-PRG filler incorporation within an H_2_O_2_-based bleaching paste on the enamel characteristics was evaluated. The results confirmed the protective effect of S-PRG filler against H_2_O_2_-induced changes in the enamel, and this requires the rejection of the null hypothesis.

Previous studies suggested that 45S5 bioglass, when used along with H_2_O_2_ bleaching solution, was beneficial for teeth whitening treatments in terms of enamel color change, microhardness, and morphological evaluation [19,20]. The 45S5 bioglass is capable of releasing Ca^2+^, Na^+^, and PO_4_^3−^ ions, resulting in the formation of hydroxycarbonate apatite, which resembles biominerals [20]. These studies indicated that 45S5 bioglass has the potential to prevent and repair enamel defects induced by bleaching agents.

Similar to 45S5 bioglass, S-PRG filler is one of the newest innovations in the category of dental materials for protecting tooth structures. This filler is designed to exhibit the inherent ability to release multiple ions that provide bioactive functions, including acid buffering, remineralization, and demineralization inhibition [23]. The applications of S- PRG filler technology within dental materials have been expanding dramatically. However, there is no evidence for its use in dental bleaching products; hence, the current study was conducted.

We used bovine teeth to examine the effect on certain enamel characteristics of the addition of S-PRG filler to a bleaching product. Although the use of human teeth reflects a more clinically relevant condition, their use is not without disadvantages, including difficulties in collecting an adequate number of sound teeth that have minimum variations in regard to the subjects from which they were collected. Moreover, the dimensions of human teeth do not allow for the creation of sufficient equally sized samples. Hence, the use of bovine teeth has been suggested as a viable alternative to their human counterparts in dental research [28].

The present study utilized plain H_2_O_2_-based bleaching paste and created enamel surfaces with high roughness, decreased microhardness, and compromised resistance to acid attack, and thus susceptible to erosion. Our findings are in accordance with studies suggesting that high concentrations of the oxidizing agent H_2_O_2_ have significant interactions with different enamel components, leading to alterations in its properties [29]. These changes are partly promoted by the effect of free radicals generated by H_2_O_2_ on the organic and inorganic matrices of enamel [30,31]. The pH of the bleaching product is another concern because of its effect on enamel demineralization, as well as other undesirable changes to the enamel surface [32]. The pH of bleaching products is set low to stabilize the H_2_O_2_ molecules and improve the shelf-life for the convenience of the manufacturers [10,33]. In fact, Akahane et al. reported that the average pH of the bleaching product used in this study was 4.8, which is well below the 5.5 critical pH of enamel hydroxyapatite [27]. Therefore, adjusting the pH of the product has been one of the interventions proposed to ameliorate the effects of the whitening procedure on tooth structure [31,33].

However, the adverse effects of H_2_O_2_ remain a controversial matter. For instance, in their work, Cadenaro et al. reported no enamel alterations [34], although they used highly concentrated H_2_O_2_ for extended application times. Thus, the authors concluded that the whitening procedure would not modify the enamel’s surface roughness. Cadenaro et al. used a neutral pH (7.0) bleaching agent that contained fluoride and potassium nitrate, which explains the absence of changes in the bleached enamel [34]. A direct comparison between the studies is not possible, as several differences should be noted, such as the pH of the used agent, as well as the assessment methods.

In the current study, we attempted to incorporate S-PRG filler into H_2_O_2_-based bleaching paste and to evaluate its effect on mitigating alternations of the enamel surface. Our previous report using S-PRG filler within a highly concentrated bleaching paste showed that S-PRG filler elevates the pH, accelerates the reaction rate of H_2_O_2,_, and enhances the whitening effect of the product [27]. The current investigation is a continuation of our previous research, and it was planned to further analyze the effect of S-PRG filler on enamel characteristics, namely, roughness, microhardness, morphological and elemental assessment, as well as resistance to acidic challenge. The current findings provide further substantiation regarding the concept of using S-PRG filler in bleaching products. We report herein the significant protective effects of S-PRG filler against H_2_O_2_-induced changes in the enamel. The positive impact of S-PRG filler may be attributed to its capacity of releasing multiple ions, which may contribute to the protective effects through different mechanisms. The pH of the bleaching product is modulated by the action of the silicate, fluoride, strontium, boron, or sodium ions [27]. Moreover, applying fluoride on the enamel surface may have rendered the surface more resistant to deleterious changes by the action of H_2_O_2_ through the conversion of hydroxyapatite to fluorapatite, which has a lower critical pH compared to that of the former. Indeed, S-PRG filler-based materials have been described to possess the smart behavior of releasing fluoride when it is most needed, specifically in acidic environments [35]. Thus, using fluoridated bleaching gels was previously purported to decrease the occurrence of undesirable changes in tooth structure without compromising the whitening efficacy [36,37]. However, controversy still surrounds the role of fluoride in bleaching agents, as a few studies found no added benefits to its inclusion in the bleaching protocol [38,39].

According to a previous study, the protective action of S-PRG filler lies not only in the mere presence of fluoride ions, but also in the presence of strontium, which contributes to creating a surface that is resistant to dissolution by the formation of strontium apatite [35]. There has been a renewed interest in the role of strontium in dental enamel after years of hiatus [40]. The intriguing properties of this trace element attracted significant attention due to its proposed ability to decrease enamel solubility, particularly in the presence of fluoride. However, to date, there is no clear understanding of the protective effect of strontium, and thus, further research is needed to elucidate its functions. In addition to fluoride and strontium ions, borate released from the S-PRG filler was recently found to adsorb to the surface of enamel and protect the substrate against demineralization [21].

Surface roughness is a frequently used parameter for evaluating the effect of bleaching products on enamel, as it is an important measurement that determines the propensity of a surface to adhere to foreign materials. Greater enamel roughness generally reflects an increased surface area and favors the adhesion and accumulation of food debris and biofilm formation [41]. The maintenance of tooth color after bleaching is an important aspect of the procedure and is one of the initial inquiries of patients seeking treatment. However, unfortunately, tooth discoloration relapses due to stain uptake over time. The results of the present study revealed that enamel bleached with S-PRG filler-incorporated paste exhibited lower roughness than that treated with plain paste, with values comparable to those of the non-bleached group. Microhardness, similar to roughness, is a key characteristic of hard dental tissue, and the knowledge of this mechanical property is paramount when developing and studying the effect of new dental materials. Enamel hardness directly relates to mineral loss or gain from the surface, consequently affecting the remineralization ability and susceptibility of the substrate to caries [42,43]. The S-PRG filler maintained the hardness of the enamel at levels comparable to those of the non-bleached group. We previously reported that 5% and 10% S-PRG filler elevated the pH of the used bleaching product from 4.8 to 6.7 and 6.8, respectively [27], and this probably mitigated the effect on roughness and hardness.

In our study, the alteration of the morphology of surfaces bleached without S-PRG filler was consistent throughout the surface, which demonstrated the surface dissolution and removal of the smear layer. These observations were suggestive of the modified physical and mechanical properties of the enamel. As described earlier, morphological changes induced by bleaching agents are not only pH-related, but are also a result of the interaction of peroxide and free radicals with organic and inorganic structures on the surface, or even subsurface, of the substrate, as these molecules have low molecular weight and thus, can penetrate the enamel [44]. Previously, it was shown that surface changes of dental enamel in the form of porosities, exposure of enamel prisms, and loss of surface smoothness are dependent on the type, concentration, and application time of the bleaching agent [45]. Also, this altered enamel is believed to be vulnerable to subsequent staining over time. Thus, smoothening enamel surfaces with polishing paste after in-office bleaching has been recommended for enhanced color stability [46]. In the current SEM investigation, the enamel morphology in specimens bleached with a paste containing S-PRG filler reflected surfaces with fewer morphological alterations, and the results were comparable to those for the non-bleached surfaces. This clarifies that the use of S-PRG filler would eliminate the need for the polishing step, thus resulting in the conservation of tooth structure and decreased post-treatment sensitivity.

The use of highly concentrated bleaching agents has been linked to an increased susceptibility of the enamel to erosion. Indeed, exposing bleached enamel to further challenge by extrinsic acids might promote demineralization that would be more pronounced than that of a non-bleached counterpart [36,47]. Owda and Sancakli pointed out the importance of contemporary non-invasive treatment options, in conjunction with bleaching, to halt the further demineralization of the bleached tooth structure [48]. Ogura et al. raised a concern regarding the lack of detailed clinical guidelines that ensure the maintenance of structural integrity of the enamel after the bleaching procedure [49]. The S-PRG filler technology is a promising option in this regard. In our study, the choice of citric acid to expose the enamel to acidic challenge was based on the fact that it is one of the acids that is most commonly added to beverages to give a tangy flavor and to preserve the product. Citric acid has a great potential to cause erosion of tooth structure due to its chelating and softening properties. The citric acid’s molecular shape and three carboxylic groups give it an immense ability to chelate with calcium and release hydrogen ions. A total of 1% citric acid at a pH of 2.1 represents the extremely erosive category of acidic beverages [50,51]. Citric acid-induced changes of enamel bleached without the S-PRG filler were more enhanced than those of the enamel bleached with the S-PRG filler-incorporated bleaching paste. As mentioned above, the incorporated fluoride and strontium ions derived from S-PRG fillers on the enamel surfaces provided significant protection to minimize the demineralization effect of the acid.

As previously reported, there was no significant difference in the bleaching effect and the pH of the H_2_O_2_ solution between the 5% S-PRG and 10% S-PRG groups [27]. In the current study, there were no significant differences between the 5% and 10% S-PRG groups in any of the experimental parameters, including surface roughness, Vickers microhardness, and the identification of the elective element by EDX. Despite the limitations in predicting actual outcomes in a complex oral environment, it may be concluded that a 5% formulation is sufficient for achieving the desired effects.

## 5. Conclusions

This in vitro study confirmed the protective effect of S-PRG filler against H_2_O_2_-induced changes on tooth enamel. With the addition of both concentrations (5% and 10%) of S-PRG filler to the bleaching paste, the roughness and microhardness of the bleached enamel surface became comparable to those of the unbleached enamel surface. Moreover, the S-PRG filler maintained the topography of the enamel and deposited fluoride and strontium ions onto the enamel surface, as revealed by the SEM and EDX assessments. The acid resistance of the enamel was also improved by the addition of the S-PRG filler to the bleaching paste. These results indicate the beneficial effects of adding S-PRG filler to bleaching products.

## Figures and Tables

**Table 1:** List of materials used and their compositions and manufacturers.

Product and Manufacturer	Composition
Shofu Hi–Lite, Shofu Inc., Kyoto, Japan	Powder: potassium compound, manganese sulphate, hydrated amorphous silica, organic copolymer, green dye Liquid: 35% hydrogen peroxide
Surface pre-reacted glass-ionomer filler, Shofu Inc., Kyoto, Japan	S-PRG filler (filler size 1 µm) Lot. BFFG-421

## Data Availability

The data presented in this study are available on request from the corresponding author.
